# Copper-caused oxidative stress triggers the activation of antioxidant enzymes via ZmMPK3 in maize leaves

**DOI:** 10.1371/journal.pone.0203612

**Published:** 2018-09-17

**Authors:** Jianxia Liu, Jinxiang Wang, Shaochin Lee, Riyu Wen

**Affiliations:** 1 School of Life Science, Shanxi Datong University, Datong, PR China; 2 School of Life Sciences, Jiangsu Normal University, Xuzhou, PR China; 3 Maize Research Institute, Shanxi Academy of Agricultural Sciences, Xinzhou, PR China; Estacion Experimental del Zaidin, SPAIN

## Abstract

Copper (Cu) is a necessary trace element participated in many physiological processes in plants. But excessive Cu^2+^ is toxic, which can activate intracellular signals that lead to cellular damage. The mitogen-activated protein kinase (MAPK) cascade is at the center of cell signal transduction and has been reported to be involved in stress-related signaling pathways. ZmMPK3, a kind of MAPKs in maize cells, can be activated by diverse abiotic stresses. In the present study, we investigated the effects of Cu^2+^ on hydrogen peroxide (H_2_O_2_) level, ZmMPK3 activity as well as the activities of antioxidant enzymes superoxide dismutase (SOD), catalase (CAT) and ascorbic acid peroxidase (APX) using maize leaf as an experimental model. The results demonstrated that acute Cu^2+^ exposure for 24 hours led to rapid increases of H_2_O_2_ level and the increase in ZmMPK3 activity as well as the total activities of antioxidant enzymes SOD, CAT and APX. H_2_O_2_ scavenger, dimethylthiourea (DMTU), effectively inhibited the Cu^2+^-increased H_2_O_2_ level and the activity of ZmMPK3 as well as the activities of the antioxidant enzymes SOD, CAT and APX. Pre-treatment with the MAPK inhibitor, PD98059, significantly blocked the Cu^2+^-increased activities of ZmMPK3, CAT, APX and SOD, but didn’t affect the accumulation of H_2_O_2_. Our results suggest that Cu^2+^ causes oxidative stress to the maize leaves which then activates defense antioxidant enzymes via MAPK pathway. Thus, the signaling pathway is Cu^2+^—H_2_O2—ZmMPK3—antioxidant enzymes.

## Introduction

Copper (Cu) is an essential trace element in plants, which participates in many physiological processes such as electron transport in photosynthesis and respiration, detoxication, and redox reaction [1]. Due to its widespread use as a pesticide and mining as well as smelting activities, the level of Cu^2+^ in the soil is often elevated. Plants absorb Cu^2+^ from the soil through the root, which further reach the aboveground part of plants through the xylem vessels. Finally, these ions are sequestered in the cell walls, vacuoles and the Golgi apparatuses through membrane transporter carriers [2]. For most plants, high concentrations of Cu^2+^ are toxic, which can cause toxicity symptoms, severe root damage and plant growth inhibition [3]. Alaoui-Sossé et al. [4] and Atha et al. [5] reported that Cu^2+^ stress can alter the ion distribution of calcium, potassium and magnesium in the cucumber root and leaves, and inhibit leaf expand and photosynthesis. Excess Cu^2+^ is also found to induce lipid peroxidation and promote potassium ion efflux in Arabidopsis seedlings [6].

Plants initially recognize heavy metal stress, activate/product signaling molecules and trigger the intracellular signal transduction, thereby mediating physiological and biochemical changes. Signaling molecules such as phytohormones, reactive oxygen species (ROS) and nitric oxide, regulate plant responses to heavy metal via gene expression [7,8], detoxification protein synthesis [9] and enzyme activity changes [2,10,11]. Hydrogen peroxide (H_2_O_2_) is thought to be a universal signaling molecule in the cell. Its rapid production plays an important role in heavy metal-induced signal pathway, which promotes the expression of antioxidant genes and enhances the capacity of antioxidant defense systems [12,13].

In all eukaryotes, the mitogen-activated protein kinase (MAPK) cascade is a universal module of signal transduction, serving at the center of intracellular signal transduction. Diverse signal pathways use MAPKs to regulate a variety of cellular functions in response to different extracellular stimuli [14–16]. There is abundant evidence that plant MAPKs can be activated by a variety of metals and play an important role in response to the metals such as AtMPK3 and AtMPK6 in arabidopsis [13], four distinct MAPKs in alfalfa, OsMPK3 and OsMPK6 in rice [17–19], and ZmMPK5 in maize [20]. A MAPK, named ZmMPK3 of group A in maize, shares high identity with the above MAPKs. Our previous studies have found that ZmMPK3 involved in diverse stress responses. Drought, oxidative stress, hormone and cadmium stress can change the transcription level of ZmMPK3 in maize [21]. In-gel kinase assay confirms that ZmMPK3 is activated by oxidative stress in maize leaves. However, the effects of Cu^2+^ on the kinase activity of ZmMPK3 in maize leaves remain poorly understood. The relationship between ZmMPK3 activation and antioxidant enzymes activities during Cu^2+^-induced stress responses has also not been well examined. In this study, we examined the relationships amongst Cu^2+^ treatment, oxidative stress, ZmMPK3 and antioxidant enzymes in maize leaf, to delineate a signaling pathway activated by Cu^2+^.

## Materials and methods

### Plant materials and design

Maize (*Zea mays* L. cv. Nongda 108) seeds were incubated and grown hydroponically in the square plastic pot (30 cm × 20 cm) filled with 1 L Hoagland solution (0.156 μM Cu^2+^) in a light chamberunder a light intensity of 200 μmol m^-2^ s^-1^ and a 14 h: 10 h (28 ˚C: 22 ˚C) day: night regimes. There are 30 seedlings in each pot. The solution was changed every 2 d.

When the second leaves were fully expanded, the seedlings were exposed to a series of the concentration of Cu^2+^ solution (0, 10, 50 and 100 μM) respectively, for 24 h at 25 ^o^C under a continuous light intensity of 200 μmol m^-2^ s^-1^. Two replicates were prepared for each concentration. There are 30 plants in each trait. To test H_2_O_2_ level, the roots of the maize seedlings were immersed into 1 mg·mL^-1^ solution of 3,3-diaminobenzidine (DAB) (pH 3.8) for 8 h under light at 25 ^o^C, and then were exposed to 100 μM CuCl_2_ for 0, 2, 4, 8, 12 and 24 h, respectively. To further investigate the effects of antioxidant dimethylthiourea (DMTU, 5 mM) and MAPK inhibitor (PD98059, 100 μM), the seedlings were pretreated with them separately for 8 h and then exposed to 100 μM CuCl_2_ for 24 h under the same conditions as described above. After Cu^2+^ treatments, the second leave from each seedling was sampled for analysis.

### Histochemical detection of H_2_O_2_

H_2_O_2_ accumulation in leaf tissues was measured using the DAB staining protocol according to the method by Orozco-Cárdebas and Ryan [22]. Briefly, plants were supplied through the roots with a 1 mg·mL^-1^ solution of DAB (pH 3.8) for 8 h, and then exposed to 100 μM CuCl_2_ solution. After these treatments, the second leaves were decolorized in boiling ethanol (95%) for 10 min. After cooling, the leaves were extracted at room temperature with fresh ethanol and photographed.

### Determination of H_2_O_2_ content

The level of H2O2 was analyzed by monitoring the A415 of the titanium–peroxide complex following the method described by Jiang & Zhang [23]. Absorbance values were calibrated to a standard curve generated with known concentrations of H_2_O_2_. Recovery was checked by adding various amounts of H_2_O_2_ to the leaf extracts as an internal standard.

### Protein extraction

Total protein was extracted from leaves with an extraction buffer according to the procedures described previously [20]. The protein concentration in tissue supernatant was evaluated with Bradford assay [24].

#### Antibody production and immunoprecipitation in-gel kinase activity assay

The ZmMPK3 polyclonal antibody was raised as described in Wang et al [21]. Immunoprecipitation in-gel kinase activity assay was performed using the method as described by Yu et al [25]. Briefly, protein extract (100 μg) was incubated with 5 μl of anti-ZmMPK3 polyclonal antibody overnight at 4 ^o^C in immunoprecipitaion buffer. About 20 μL packed volume of protein A-agarose was added, and the incubation was continued for another 2 h. The protein-antibody complexes on the beads were pelleted by centrifugation and washed three times with wash buffer and once with kinase buffer. Kinase activity was assayed at 30 ^o^C for 30 min in a final volume of 25 μl containing 0.5 mg·mLw^-1^ of myelin basic protein, 10 μM ATP, 10 μCi of [γ^32^P]-ATP and the beads with ZmMPK3. The action was stopped by the addition of SDS-PAGE sample loading buffer. After electrophoresis, the phosphorylated substrates were visualized by autoradiography.

### Enzyme activities assays of SOD, CAT and APX

Frozen leaf segments were homogenized and the homogenate was centrifuged and the supernatant was immediately used for the antioxidant enzyme assays. The activities of SOD, CAT and APX were determined as described previously [23]. SOD activity was assayed by monitoring the inhibition of photochemical reduction of NBT. One unit of SOD was defined as the amount of protein that inhibited the rate of NBT reduction by 50% at 560 nm. CAT activity was assayed by measuring the rate of decomposition of H_2_O_2_ at 240 nm. APX activity was measured by monitoring the decrease in absorbance at 290 nm as ascorbate was oxidized. The units of antioxidant enzymes activities were U mg^-1^ protein (SOD), μmol min^-1^ mg^-1^ protein (CAT) and μmol min^-1^ mg^-1^ protein (APX), respectively.

### Statistical analysis

All Statistical analyses were performed using SPSS 22.0 computer software package. Data were expressed as mean values ± S.E. Differences among groups were examined by one-way ANOVA followed by LSD. *P*<0.05 was considered as statistically significant.

## Results

### H_2_O_2_ production in the leaves of maize exposed to Cu^2+^

**Fig 1:**
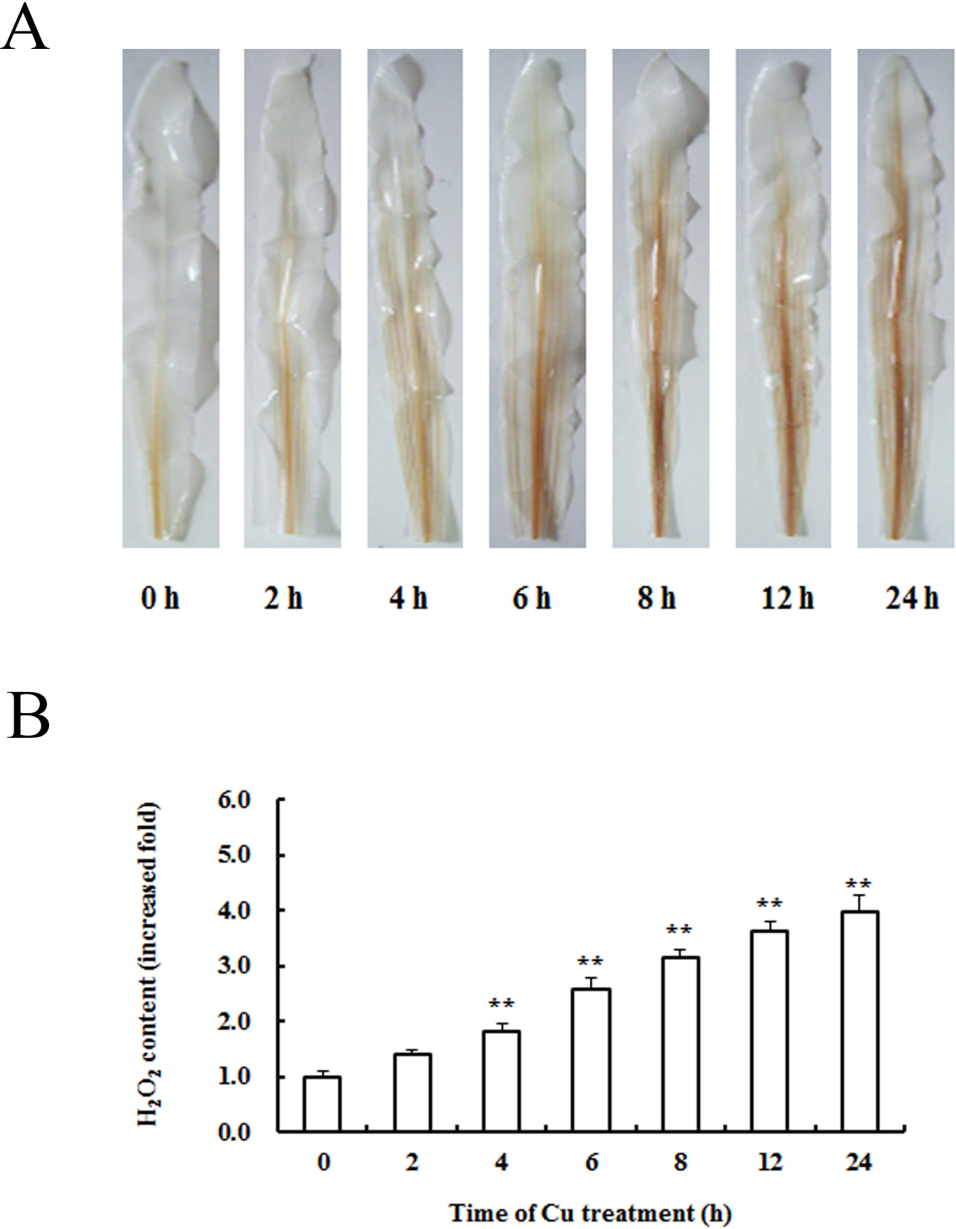
H_2_O_2_ accumulation in the leaves of maize exposed to Cu^2+^. (A) Histochemical detection of H_2_O_2_ production with DAB staining; (B) Determination of H_2_O_2_ content using spectrophotometric method. Results are presented as mean ± S.E. (n = 6) of three experiments. The mean value of the control is ascribed an arbitrary value of 1 and the mean value in each treated group is shown as a fold increase compared to the mean value in the control. The experiments were replicated three times. * denotes *P*<0.05, ** *P*<0.01.

The reaction of DAB with H_2_O_2_ can produce the deep brown polymerization product. DAB stain, a histochemical method for H_2_O_2_ detection, was employed to test H_2_O_2_ accumulation in leaves of maize plants exposed to Cu^2+^ stress. It was observed that brown polymerization products were barely seen in the base of leave in the control plants, which indicated that the level of H_2_O_2_ was low (Fig 1A). Visible H_2_O_2_ accumulation was observed in leaves of maize plants exposed to Cu^2+^ for 2 h, which was obviously seen at 4 h. Cu^2+^ led to H_2_O_2_ production in a time-dependent manner (Fig 1A). H_2_O_2_ content in leaves of maize plants were examined using the methods of spectrophotometry. Fig 1B shows that treatment with 100 μM Cu^2+^ for 2 h increased the content of H_2_O_2_ but did not change significantly compared to the control value. After 4 h of Cu^2+^ treatment, the levels of H_2_O_2_ rose significantly in a time-effect manner.

### Effects of Cu^2+^ stress on ZmMPK3 activity in maize leaves

**Fig 2:**
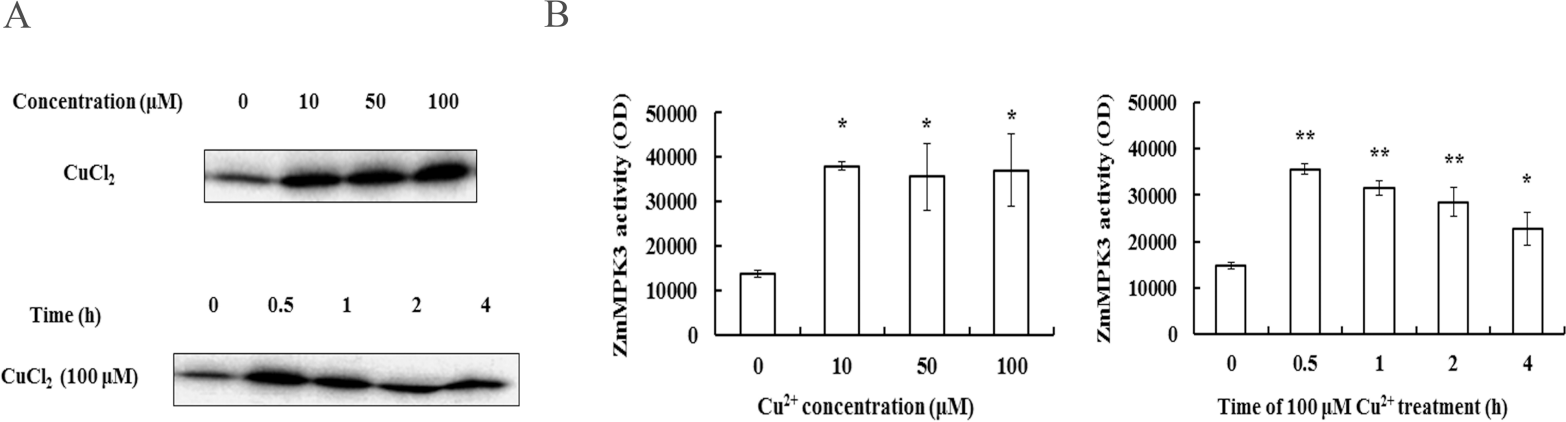
Effects of excess Cu^2+^ exposure on ZmMPK3 activity in maize leaves. (A) ZmMPK3 kinase activity. (B) Quantification of ZmMPK3 activity. In-gel images were analyzed by Image J image processing software. Data are shown as mean ± S.E. of three independent experiments. Plants were treated with various concentrations of Cu^2+^ (0, 10, 50 and 100 μM) for 0.5 h or 100 μM Cu^2+^ for different times (0, 0.5, 1, 2 and 4 h). All experiments were replicated three times. * denotes *P*<0.05, ** *P*<0.01.

To investigate the effect of Cu^2+^ on ZmMPK3 activity, the polyclonal antibody that recognizes the C-terminal region of ZmMAPK3 was raised in rabbits, and the immune-precipitation in combination with in-gel kinase assay was performed. As shown in Fig 2, Cu^2+^ treatment increased the ZmMPK3 activity in does- and time-dependent manners (Fig 2).

### Effects of excess Cu^2+^ exposure on the activities of antioxidant enzymes

**Fig 3:**
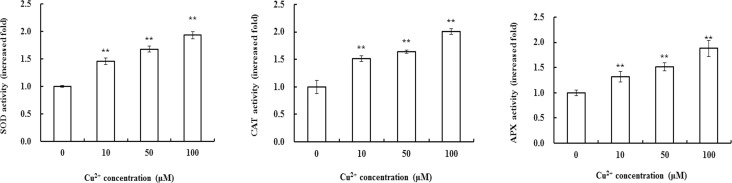
Effects of Cu^2+^ stress on the activities of SOD, CAT and APX in leaves of maize. Plants were treated with various concentrations of Cu^2+^ for 24 h. Results are expressed as mean ± S.E.(n = 6) of there different experiments. The mean value of the control is ascribed an arbitrary value of 1 and the mean value in each treated group is shown as a fold increase of that in the control. * denotes *P*<0.05, ** *P*<0.01.

High concentration of H_2_O_2_ is harmful to cells. So in the course of evolution, plants have developed a protective system that can reduce oxidative stress and damage. Enzymes SOD, CAT and APX are ROS scavengers in the anti-oxidant protection system. Therefore, we further measured the activities of these three enzymes. Excess Cu^2+^ increases the activity of SOD in leaves of maize seedling in a dose-dependent manner (Fig 3). The activity of SOD reached its maximum when treated with Cu^2+^ at 100 μM, which was approximately 200% of that in the control group. Similar to SOD activity, Cu^2+^ increased the activities of CAT and APX in a dose-dependent manner (Fig 3). And the change in the activity of the two enzymes was in the similar manner with that of SOD’s, i.e., the activities of CAT and APX increased gradually with the increase of Cu^2+^ concentration. At 100 μM, Cu^2+^ treatment increased the activities of CAT and APX to the maximum, which were 1.93 and 1.88 times of that in the control groups, respectively.

### Relationship between H_2_O_2_ production and ZmMPK3 activation induced by Cu^2+^ stress

**Fig 4:**
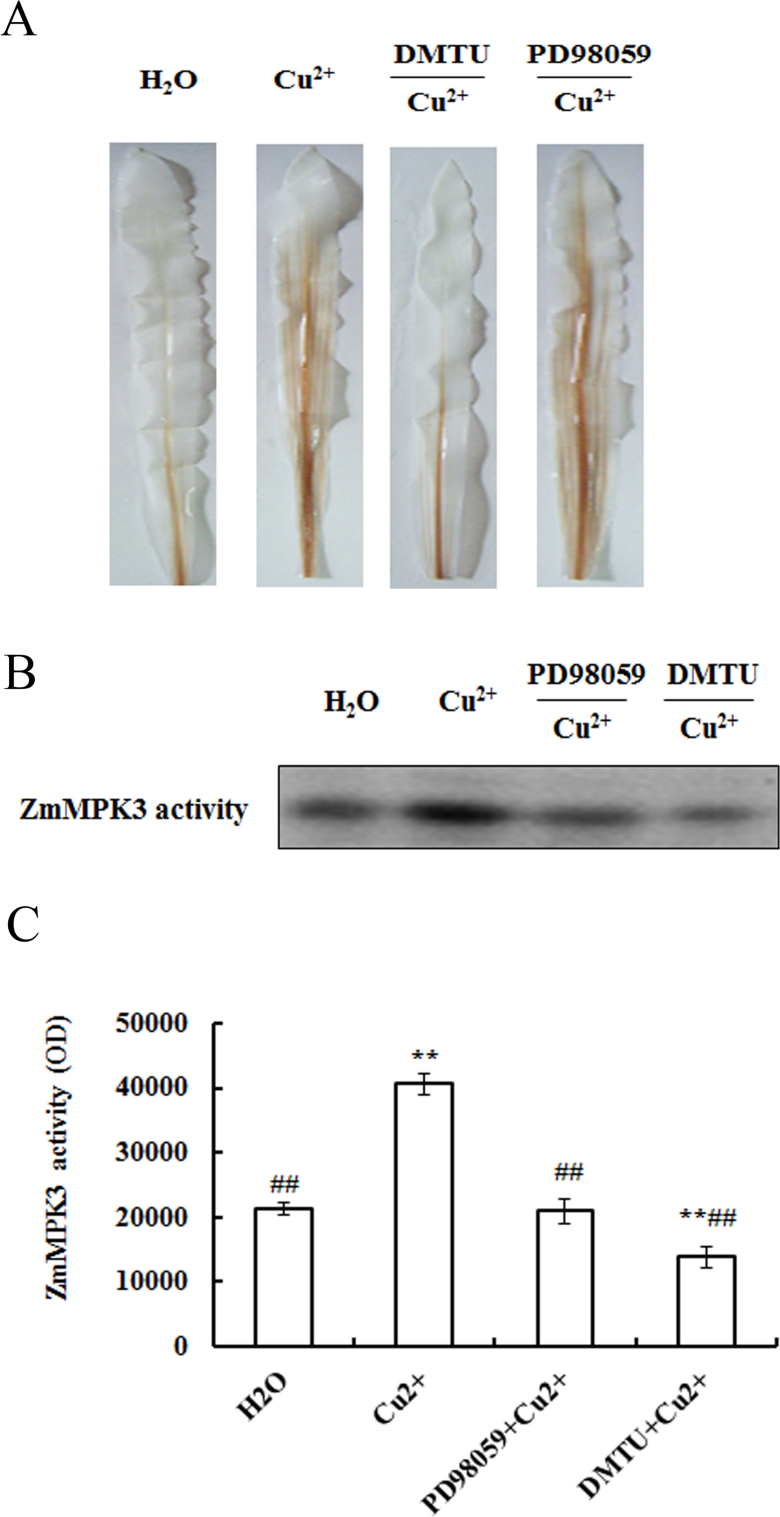
Relationship between H_2_O_2_ production and ZmMPK3 activation induced by excess Cu^2+^ in maize leaves. (A) Effects of pretreatment with PD98059 or DMTU on H_2_O_2_ production induced by excess Cu^2+^. Maize plants were pretreated with or without 100 μM PD98059/5 mM DMTU for 8 h, then exposed to 100 μM Cu^2+^ for 24 h. The letters on the lanes represent: H_2_O = H_2_O (8 h) + H_2_O (24 h); Cu^2+^ = H_2_O (8 h) + 100 μM Cu^2+^ (24 h); DMTU/ Cu^2+^ = 5 mM DMTU (8 h) + 100 μM Cu^2+^ (24 h) and PD98059/ Cu^2+^ = 100 μM PD98059 (8 h) + 100 μM Cu^2+^ (24 h). (B) Effects of pretreatment with PD98059 or DMTU on ZmMPK3 kinase activity. (C) Quantification of ZmMPK3 activity. In-gel images were analyzed by Image J image processing software. Data are shown as mean ± S.E. of three independent experiments. Maize plants were pretreated with or without 100 μM PD98059/5 mM DMTU for 8 h, then exposed to 100 μM Cu^2+^ for 0.5 h. The letters on the lanes represent: H_2_O = H_2_O (8 h) + H_2_O (0.5 h); Cu^2+^ = H_2_O (8 h) + 100 μM Cu^2+^ (0.5 h), PD98059/ Cu^2+^ = 100 μM PD98059 (8 h) + 100 μM Cu^2+^ (0.5 h) and DMTU/ Cu^2+^ = 5 mM DMTU (8 h) + 100 μM Cu^2+^ (0.5 h).The experiment was replicated three times.On comparing with the control, significance is shown by *P<0.05, **P<0.01; on comparing with Cu^2+^ treatment group, ^#^*P*<0.05, ^##^*P*<0.01.

To study the relationship between H_2_O_2_ production and ZmMPK3 activity, the DMTU, a H_2_O_2_ scavenger, and PD98059, a MAPK inhibitor, were used. Cu^2+^ treatment led to the accumulation of H_2_O_2_ in maize leaves. Pre-treatment of PD98059 inhibited the Cu^2+^-triggered ZmMPK3 activation, but didn’t inhibit the increase of H_2_O_2_ level (Fig 4A). As shown in Fig 4B and 4C, Cu^2+^ treatment led to an increase in the activity of ZmMPK3. Pre-treatment of DMTU almost blocked the increment in H_2_O_2_ level and ZmMPK3 activity induced by Cu^2+^ stress.

### Effects of pre-treatment with DMTU or PD98059 on antioxidant enzymes activities induced by Cu^2+^ stress

**Fig 5:**
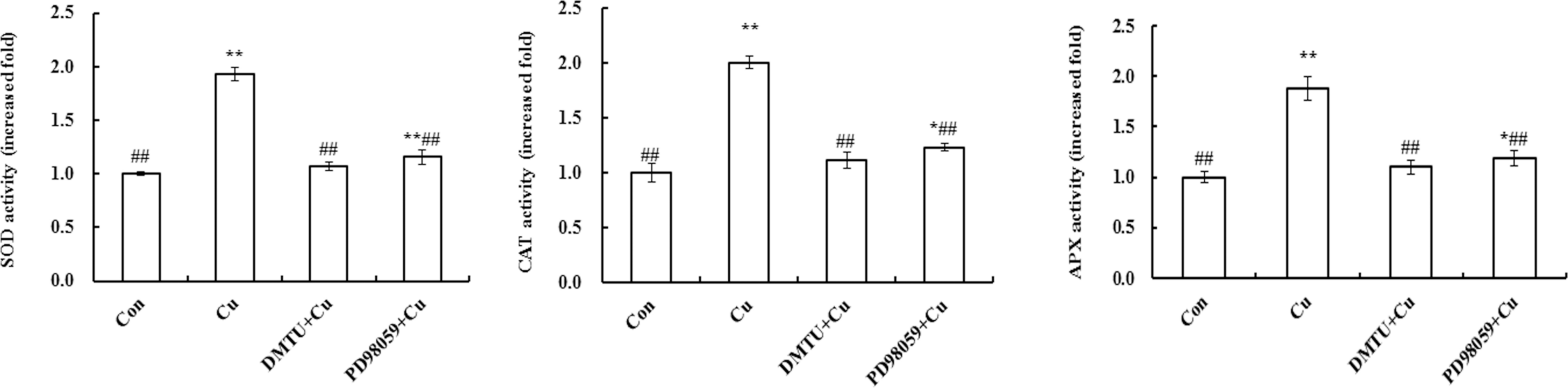
Effects of pretreatment of DMTU or PD98059 on the activities of SOD, CAT and APX in maize leaves. The maize plants were pretreated with or without 5 mM DMTU or 100 μM PD98059 for 8 h, then exposed to 100 μM Cu^2+^ or distilled water for 24 h. The letters on the lanes represent: Con = H_2_O (8 h) + H_2_O (24 h); Cu = H_2_O (8 h) + 100 μM Cu^2+^ (24 h), DMTU+Cu = 5 mM DMTU (8 h) + 100 μM Cu^2+^ (24 h) and PD98059+Cu = 100 μM PD98059 (8 h) + 100 μM Cu^2+^ (24 h). Results are presented as mean ± S.E.(n = 6) of three experiments. The mean value of the control is ascribed an arbitrary value of 1 and the mean value in each treated group is shown as a fold increase compared to the mean value in the control. All experiments were replicated three times. On comparing with the control, significance is shown by *P<0.05, **P<0.01; on comparing with Cu^2+^ treatment group, ^#^*P*<0.05, ^##^*P*<0.01.

Fig 5 showed that the activities of SOD, CAT and APX increased significantly in the leaves after being treated by excess Cu^2+^ compared with that in the controls. But the increases of antioxidant enzymes activities were suppressed by DMTU or PD98059.

## Discussion

The earlier period of the exposure to a stress factor is critical and it will determine further changes in the organism. During this period some signaling pathways are activated, which may enhance the resistance or/and aggravate the stress [26]. Cu is a transition metal with an electrochemical potential and participates in important redox reactions in cellular electron transport chains, for example as a cofactor of oxidases [1]. But large doses of Cu^2+^ is acutely toxic for all plants [4,13]. One of Cu^2+^ toxicity is to catalyze the formation of ROS [27]. However, as ubiquitous signaling molecule, ROS also involved in the recognition of and the response to stress factors, influencing signal transduction and gene expression [28]. In this study, the oxidative-redox state of maize leaves after Cu^2+^ treatment was investigated using the DAB staining and spectrophotometric method, respectively. The results showed that Cu^2+^ exposure led to H_2_O_2_ productions in a short period (e.g. 2 h), and the H_2_O_2_ accumulation was enhanced with the prolong time. The results were consistent with the findings of Hu et al. [29], Maksymiec and Krupa [26], which demonstrated that the level of H_2_O_2_ and O_2_^•—^ increased markedly during the first hours of excess Cu^2+^ treatment in maize and arabidopsis leaves. In a similar line of evidence it was shown that exposure to excess Cu^2+^ caused increases of ROS level in purpurea and rice [19, 30]. It can be seen that the rapid production of H_2_O_2_ is an early response of plants to Cu^2+^ stress.

MAPK cascade has been shown to be associated with signaling transmission from cytoplasm to nucleus, and plays a central role in the expression of resistance-related genes [11]. The interaction of Cu^2+^ with MAPK seems to be an important parameter to explore the mechanism of a possible detoxification effect of Cu^2+^. In plant, convincing evidence demonstrates interference of Cu^2+^ with MAPKs. Exposure of alfalfa (*Medicago sativa*) seedlings to excess Cu^2+^ rapidly activated four distinct MAPKs including SAMK, SIMK, MMK2, and MMK3 [17]. In rice it activated, at least, three different MAPKs, including OsMPK3, OsMPK6, and 40 kDa MAPK, which regulate heavy metal stress tolerance [19]. In the present study, Cu^2+^ stress induced the increase of ZmMPK3 activity in a relatively short time (e.g. 0.5–4 h), which suggested that ZmMPK3 signal pathway was activated by Cu^2+^ and involved in stress response to heavy metal (Fig 2). It transmits signals through phosphorylation, which ultimately activates effector proteins or promotes transcription of resistance-related genes [11,22].

Numerous changes that occur under stresses include both pathological consequences of stress injury and adaptive responses [30]. Metal ions exposure can increase ROS production. High concentration of ROS is harmful to cell, which causes a series of pathological changes, such as lipid peroxidation, membrane damage and enzymes inactivation as well as cell viability [31,32]. In order to avoid the diverse effects from ROS, plants have formed an antioxidant network and trigger adaptive responses. Antioxidant enzymes (such as SOD, CAT and APX) involved in ROS scavenging [26,33]. O_2_^•—^ scavenging by SOD and H_2_O_2_ decomposition by APX and CAT are mainly related to the maintenance of cellular redox stability. The rapid O_2_^•—^ generation occurred concomitantly with enhanced SOD activities in the Cu^2+^-treated (1–6 h) wheat roots [34]. Cu^2+^ tolerance in pea correlated with increased activities of SOD and CAT. Lombardi and Sebastiani [35] reported that Cu^2+^ stress increased total CAT and SOD activity and induced simultaneously SOD and CAT gene expression in *Prunus cerasifera*. In the present study, it is interesting to note that H_2_O_2_ rapid accumulated in excessive Cu^2+^-treated seedling. In view of this, the activities of three antioxidant enzymes (SOD, CAT and APX) in maize leaves exposed to Cu^2+^ stress were analyzed (Fig 3). The data showed that their activities were increased significantly at 24 h of Cu^2+^ treatment, indicating that Cu^2+^ exposure increased the content of ROS in plants, but at the same time, it also activate the defense system. Plants reduced the cellular ROS level and weakened cytotoxicity induced by Cu^2+^ through enhancing antioxidant enzymes activities. Our observation was consistent with that of Hu et al. [29], which indicated that Cu^2+^ led to the increase of antioxidant enzymes activities in maize leaves. Excess Cu^2+^ can increase content and/or activity of antioxidants which contribute to remove "free" Cu^2+^ and to re-establish cellular ion and redox homeostasis. So, the increase of antioxidant enzymes activities is a kind of detoxification responses, which reduce stress injury caused by Cu^2+^ and improve stress tolerance.

We further focused our attention on the relationships among H_2_O_2_, ZmMPK3 and antioxidant enzymes under Cu^2+^ stress. Our previous work has shown that the transcription level and activity of ZmMPK3 in maize seedlings were increased after being exposed to H_2_O_2_ [21]. Many studies showed that heavy metals-induced ROS production plays an important role in MAPK activation [17,36,37]. ZmMPK5 in maize was activated by both drought and ABA, which is regulated by H_2_O_2_ [22,38]. The activities of OsMPK3 and OsMPK6 in rice were induced by both Cd^2+^ and Cu^2+^, and the process is associated with ROS [19]. MAPK pathways integrate diverse signaling stimuli. So we hypothesized that H_2_O_2_ induced by Cu^2+^ maybe involved in ZmMPK3 activation. To further investigate the regulation of ROS on MAPK pathway, maize seedlings were pretreated with DMTU (H_2_O_2_ scavenger) before Cu^2+^ exposure and ZmMPK3 activity was analyzed. The result showed that pretreatment of DMTU inhibited the activation of ZmMPK3 induced by Cu^2+^. But PD98059 pretreatment didn’t affect H_2_O_2_ production (Fig 4). It is clear that H_2_O_2_ is an essential regulator of ZmMPK3 activation under Cu^2+^ stress. This result was similar to that of Yeh et al. [19], who reported that Cu^2+^ stimulates MAPKs activation via ROS generation and each MAPK activation depends on different types of ROS in rice roots. Plants also use ROS in signal transduction cascades inducing defense responses [39]. MAPKs cascades controlled H_2_O_2_-induced defense reaction [40]. Mattie and Freedman [11] reported that excess Cu^2+^ influenced metallothionein expression through activation of MAPK signaling pathway to reduce the toxicity of heavy metal. We found, in the present study, that Cu^2+^ stress led to ROS production and ZmMPK3 activation, and ZmMPK3 activation depended on the generation of ROS. In addition, Cu^2+^ stress also increased the activities of three antioxidant enzymes and improved the defense capability of the plant. But the relationships of MAPK activation, H_2_O_2_ production and antioxidant defense has not been yet studied. We speculate that a Cu^2+^- H_2_O_2_-MAPK-antioxidant defense signal pathway may exist in maize. We next investigated whether the H_2_O_2_ and ZmMPK3 was essential for antioxidant defense induced by Cu^2+^ stress in maize. To address this question, DMTU and PD98059 were used in the study. Pretreatment of the two inhibitors attenuated the increases of three antioxidant enzymes activities induced by Cu^2+^ stress (Fig 5). The results indicated that Cu^2+^ stress led to ROS production, which activated MAPK pathway including ZmMPK3 signal pathway. Phosphorylated ZmMPK3 resulted in an increase in antioxidant enzymes activity. H_2_O_2_ and activated MAPK signal protein by Cu^2+^, as the upstream input signals of detoxification responses, regulated the activity of antioxidant enzymes and improved the stress tolerance.

## Conclusions

In summary, this study clearly demonstrated that exposure to excess Cu^2+^ induced H_2_O_2_ accumulation and led to oxidative stress in the maize leaves. H_2_O_2_, a production of cell injury, activated the MAPKs cascade system and caused ZmMPK3 activation. Plants enhanced the antioxidant ability through the increase of antioxidant enzymes activities to reestablish cellular redox homeostasis under stress conditions and cope with Cu^2+^-induced oxidative stress. H_2_O_2_—ZmMPK3 signal pathway initiate adaptive responses, e.g., antioxidant responses, which in turn alleviate the cytotoxicity caused by Cu^2+^. Thus, the signaling pathway is Cu^2+^—H_2_O2—ZmMPK3—antioxidant enzymes.
